# Quantitative Evaluation in Estimating Sources Underlying Brain Oscillations Using Current Source Density Methods and Beamformer Approaches

**DOI:** 10.1523/ENEURO.0170-19.2019

**Published:** 2019-08-05

**Authors:** Tamesh Halder, Siddharth Talwar, Amit Kumar Jaiswal, Arpan Banerjee

**Affiliations:** 1 Cognitive Brain Dynamics Lab, National Brain Research Centre, NH8, Manesar, Haryana 122052, India

**Keywords:** DICS, EEG, eLORETA, LCMV, MEG, MNE

## Abstract

Brain oscillations from EEG and MEG shed light on neurophysiological mechanisms of human behavior. However, to extract information on cortical processing, researchers have to rely on source localization methods that can be very broadly classified into current density estimates such as exact low-resolution brain electromagnetic tomography (eLORETA), minimum norm estimates (MNE), and beamformers such as dynamic imaging of coherent sources (DICS) and linearly constrained minimum variance (LCMV). These algorithms produce a distributed map of brain activity underlying sustained and transient responses during neuroimaging studies of behavior. On the other hand, there are very few comparative analyses that evaluates the “ground truth detection” capabilities of these methods. The current article evaluates the reliability in estimation of sources of spectral event generators in the cortex using a two-pronged approach. First, simulated EEG data with point dipoles and distributed dipoles are used to validate the accuracy and sensitivity of each one of these methods of source localization. The abilities of the techniques were tested by comparing the localization error, focal width, false positive (FP) ratios while detecting already known location of neural activity generators under varying signal-to-noise ratios (SNRs). Second, empirical EEG data during auditory steady state responses (ASSRs) in human participants were used to compare the distributed nature of source localization. All methods were successful in recovery of point sources in favorable signal to noise scenarios and could achieve high hit rates if FPs are ignored. Interestingly, focal activation map is generated by LCMV and DICS when compared to eLORETA while control of FPs is much superior in eLORETA. Subsequently drawbacks and strengths of each method are highlighted with a detailed discussion on how to choose a technique based on empirical requirements.

## Significance Statement

State-of-the-art methods of source localization techniques, e.g., current density methods, minimum norm estimates (MNE) and beamformers report distributed brain activity patterns that are often not in consensus for a putative task. This article offers ground truth validation of these techniques in the context of different kind of source detections, e.g., determining the sources underlying key events (evoked potentials) and steady state brain oscillations (band limited brain activity). The broader goal is to help cognitive neuroscientists select the most effective source localization technique that is in sync with the signal processing needs required for targeting a specific question.

## Introduction

Cortical oscillations play an important role in governing basic cognitive functions (Edelman and Mountcastle, 1978; Bressler and Kelso, 2001; Buzsáki and Draguhn, 2004). Several researchers have suggested that electromagnetic brain activity at specific frequency bands carries meaningful information about neural function, e.g., alpha waves at 10 Hz (Llinás et al., 1999; Bollimunta et al., 2008), beta at 15–30 Hz (Brovelli et al., 2004), and gamma at 30 Hz and above (Bressler et al., 1993; Varela et al., 2001; Cheyne and Ferrari, 2013). Concurrently, time-locked transient responses have been useful for decades in electrophysiological research, both for understanding basic neurobiological functions as well as in clinical and other applications (Picton et al., 1974; Kutas et al., 1977; Clark et al., 1994; Pantev et al., 1995; Cheyne et al., 2006). Hence, identifying the neural generators of sustained cortical oscillations and task-specific transient neural responses from EEG/MEG is an extensive topic of research. Once identified with adequate reliability, the focal localization of sources will eventually reveal the underlying large-scale network governing cognitive tasks.

There are several source localization methods in the literature, commonly known under the umbrella of inverse methods (Hämäläinen and Sarvas, 1989). Most of these techniques are based on fitting single/multiple dipolar cortical source/sources within a defined cortical volume based on some assumptions about relationships between the sources (Hämäläinen and Ilmoniemi, 1994; Van Veen et al., 1997; Ishii et al., 1999; Gross et al., 2001; Liu et al., 2002; Hillebrand and Barnes, 2003; Sato et al., 2004). Some methods consider sources to have minimum correlation, e.g., synthetic aperture magnetometry (SAM; Hillebrand and Barnes, 2003), linearly constrained minimum variance (LCMV) spatial filtering (Van Veen et al., 1997; Murzin et al., 2011). There are specialized measures that detect generators of oscillatory brain signals by considering maximum coherence between prospective sources, e.g., dynamic imaging of coherent sources (DICS; Gross et al., 2001) and entropy based metrics (Lina et al., 2014). DICS is a frequency domain extension of beamforming methods over the initially developed time-domain beamformers, e.g., LCMV and SAM (Ishii et al., 1999), which are primarily used for determining the sources underlying time-locked event-related potentials/event-related fields (ERPs/ERFs) components. In EEG, where deeper sources can affect scalp potentials, current density techniques such as minimum norm estimates (MNE; Hämäläinen and Ilmoniemi, 1994) and exact low-resolution brain electromagnetic tomography (eLORETA; Pascual-Marqui, 2007) have been the method of choice. Although dynamic statistical parametric mapping (dSPM; Liu et al., 2002) and sparse Bayesian learning (SBL; Ramírez et al., 2010) have been developed to improve on the estimates of spatial filter detection, eLORETA is still by far one of the most robust methods for EEG source localization. eLORETA directly estimates current source density, a biophysically relevant parameter over a grid of plausible cortical locations for both detection of time-locked activity, e.g., in ERP/ERF or frequency-locked activity, e.g., spontaneous frequency bursts or steady state oscillatory responses to periodic stimuli. Nonetheless, the source estimated by all methods is broadly influenced by depth, signal-to-noise strength of the neural activity, as well as the correlation in the covariance of the signals (Belardinelli et al., 2012) and redundant informational content of high temporal resolution data. Often, these manifest in distributed source activity estimation with diminished statistical power.

The accuracy of the location of neural activity along with lower false positives (FPs) should be the expectation from any source localization technique. In this article, we evaluate the performance of the key current density techniques: eLORETA and MNE, and beamformer approaches: DICS and LCMV, on simulated EEG data. Since LCMV and DICS belong to the same class of beamformers, they were compared against eLORETA, to quantify localization efficiency between beamformers versus current density measures. We compared the results from eLORETA and DICS on a paradigm of evoked 40-Hz auditory steady state responses (ASSRs) and eLORETA, MNE, and LCMV for detecting the source of N100 activity when the same data were epoched time locked to the stimulus onset. Many inverse methods can localize a transient or steady state response or both. However, the biological relevance or interpretation of these different information processing events can be very distinct. There are comparison studies that evaluate the performance across different methods (Bradley et al., 2016; Hedrich et al., 2017) or sometimes the performance of detecting a focal cortical source across modalities EEG and MEG (Srinivasan et al., 2006; Mideksa et al., 2015). In this article, our focus was to compare the specificity and sensitivity of some of the prominent algorithms primarily chosen based on their conceptual difference current density estimate versus beamforming to provide a basis for choosing one above the other when faced with the issues of transient or steady state response. Very rigorous comparison metrics, e.g., localization error, spatial spread, and FP percentage, were used to evaluate accuracy and sensitivity of results along with an evaluation of the performance of these methods at different depths of dipole placement in the simulated EEG data. Subsequently, empirical EEG data during N100 and ASSRs were used to draw comparisons among distributed nature of source activity patterns generated by these methods.

## Materials and Methods

### Generation of synthetic EEG data

**Figure 1.**
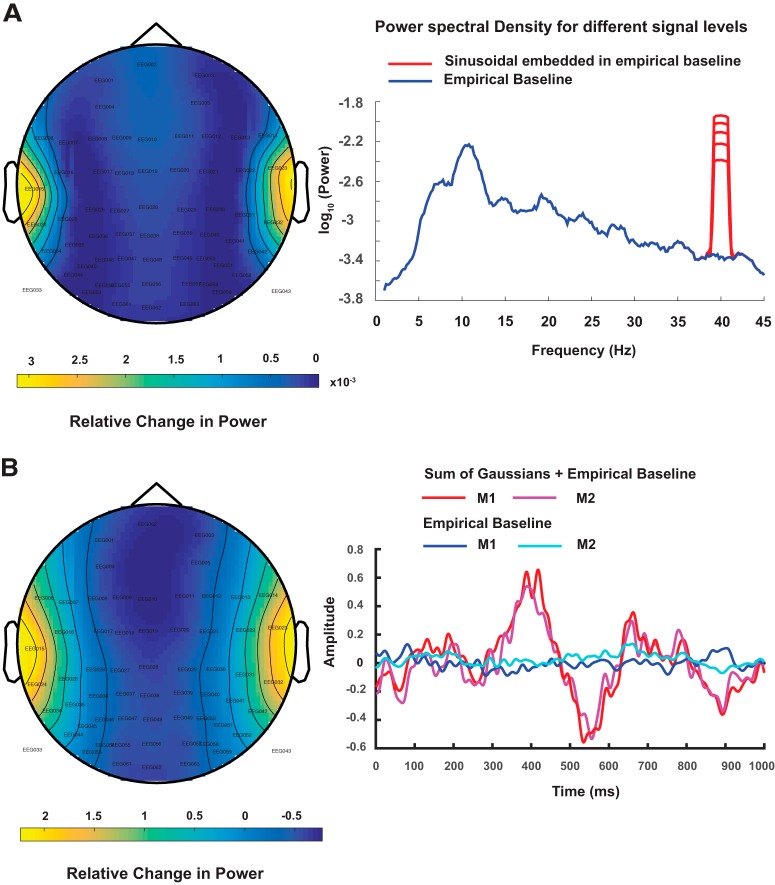
***A***, Topoplot of the difference in peak of spectral power at 40 Hz obtained from simulated EEG data where dipolar source time series was represented by a sinusoidal signal with frequency 40 Hz embedded in empirical resting state EEG and empirical resting state EEG as baseline. Spatially averaged power spectra obtained from averaging the channel-by-channel spectrum from hypothetical scalp sensors are plotted in logarithmic scale. The time series on the scalp were obtained by applying forward modeling techniques on dipolar sources at auditory cortex locations using the Boundary element method (BEM). ***B***, Topoplot of the peak of difference signal when a mixture of Gaussian pulses was used to simulate ERP and empirical resting state EEG as baseline in BEM model as described in ***A***. The time series for dipole dynamics are plotted at hypothetical M1 and M2 sensors located near to the auditory cortices. The positive peak at 400 ms was used for generating the topoplot.

To localize the oscillatory activity, as well as the transient response, we simulated a time-varying sinusoid at 40 Hz and a mixture of Gaussian pulses, respectively. Both generated signals were free of noise and were consequently added to the acquired empirical baseline, for realistic noise simulation. The magnitude of the dipolar source dynamics in cortical locations are represented by

(1) $$q_{i}(t)=sin(2\pi 40t)+\epsilon (0,\sigma ),$$

where *q_i_*(*t*) is the electric dipole moment at location *i* and at time *t*, *ϵ* is white noise with zero mean and SD *σ*. We compared three conditions with respect to number of sources, by placing single-point dipole, two-points dipoles, and distributed dipoles in a Montreal Neurologic Institute (MNI) brain template according to the MNI (Müller and Weisz, 2012). Single-point source was placed at around the superior temporal region, in the left hemisphere [MNI coordinates: (–60, –28, 6)]. Two sources were placed, one in the left hemisphere [MNI coordinates: (–60, –28, 6)] and the other in the right hemisphere [MNI coordinates: (64, –24, 6)] around the superior temporal region. Approximately, 100-point sources were placed within a spherical volume with radius of 12 mm in the left hemisphere centered around the superior temporal region at (–60, –28, 6), according to brain template. Another set of 100-point sources was placed around the right hemisphere auditory cortex seed area at (64, –24, 6), defining the distributed source condition. The resolution of the grid chosen for dipole simulation was 5 mm, and ft_prepare_leadfield.m code of FieldTrip toolbox was used for this purpose. Dipole moment orientations were assumed to be along the radial direction with respect to the BEM surface, to retain simplicity. We computed the scalp potentials for EEG at realistic sensor locations by applying a forward model (Mosher et al., 1999; Baillet et al., 2001) with realistic headshape using ft_dipolesimulation.m of the FieldTrip toolbox.

(2) $$v_{r}(t)=L^{T}(r,r_{q}).q(t),$$

where *v* is the electric potential at sensor location *r*, *r_q_* represents all source locations, **L** represents the “lead field kernel,” (.)*^T^* represents transpose, and *q*(*t*) is the dipole moment. Synthetic EEG data were generated by varying signal-to-noise ratio (SNR) at the source space. Physiologic SNR was estimated using a statistical measure, $10\log_{10}[\frac{s}{\sigma_{b}}]$, where *s* is peak-to-peak amplitude of EEG data during rhythmic auditory stimulation (see experimental methods below), and *σ_b_* is the SD of the baseline data. We chose a wide range of SNRs (19, 22, 25, 28, 31 dB) to simulate mixture of Gaussian pulses mimicking transient response, both above and below the estimated physiologic SNR level (25 dB), to allow us to evaluate the sensitivity of each method. Further, we introduced time lags between the signals generated from the left and right hemispheres for two and distributed dipole models. Time delays of 0, 15, 30, 45 ms were added to the Gaussian pulse generated from the right hemisphere. Figure 1 shows simulated EEG activity on scalp surface with bilateral auditory cortical sources. Following Goldenholz et al. (2009), SNR was computed in dB using the following equation:

(3) $$SNR_{dB}=10\log_{10}[\frac{1}{U}\sum_{u=1}^{U}\frac{v_{u}^{2}}{\eta_{u}^{2}}],$$

where *U* is total sensor count, and *v* is the signal on sensor u∈(1,2,⋯U) provided by the forward model for a source with unit amplitude. The sensor space variance is expressed as $\eta_{u}^{2}=\sigma^{2}{\left(LL^{T}\right)}_{u}$. Subsequently, for each time lag scenario, we simulated the Gaussian signal of five SNR values. Additionally, we also simulated sinusoidal signals mimicking oscillatory activity with different values of power spectra at 40 Hz. The power values were chosen with respect to the power spectrum computed for the empirical binaural condition data, such that the power of simulated sinusoidal at 40 Hz was 50%, 75%, 100%, 125%, and 150% of the power at 40 Hz of the binaural condition, illustrated in Figure 1. Further, phase lags were introduced between the signals generated in left and right hemisphere: 0, *π*/2, *π*, and 3*π*/2. Therefore, all the power ratio scenarios were computed for each phase lag condition.

### Source localization methods

The basic goal of any source localization technique is to compute the dipolar source locations and strengths inside the brain from measurements on the scalp (inverse of Equation 2). In other words, the objective is to estimate the spatial filter **W***_S_* from the relation

(4) $$q(t)=W_{S}\dot{}V(t),$$

where *q*(*t*) is the dipole moment at time *t*, **W***_S_* is the spatial filter matrix, and **V** is vector representation of all sensor time series. Obviously, the system of equations represented by Equation 4 is ill-posed, as the number of sensors (dimension of vector **V**) is finite, but the number of dipoles is unknown. Thus, different source localization methods attempt to estimate the **W***_S_* using diverse constraints posed by anatomy of the brain and functional relationships among brain areas during ongoing task.

#### LCMV

LCMV belongs to the class of “beamformer” methods that enhances a desired signal while suppressing noise and interference at the output array of sensors (Barnes and Hillebrand, 2003). LCMV is built on an adaptive spatial filter whose weights are calculated using covariance matrix of EEG/MEG time series data. A spatial filter computes the variance of the total source power which is allowed to vary but the output of the filtered lead field is kept constant. As a result, the beamformer output is maximized for the target source but other source contributions are suppressed. LCMV attempts to minimize the beamformer output power

(5) $$P=W_{S}^{T}CW_{S}S$$

where **C** is the data covariance matrix. The entries to spatial filter matrix can be expressed as

(6) $$W_{S}=\frac{L^{T}C^{-1}}{L^{T}C^{-1}L},$$

where **L** is lead field matrix, and the following constraint is maintained $W_{S}\dot{}\;L^{T}=1$.

#### DICS

DICS beamformer (Gross et al., 2001) works with same constraint assumption of LCMV but extends the computation of spatial filter to the frequency domain. Here, sensor level cross-spectral density (CSD) matrix replaces the covariance matrix, and the spatial filter is applied to sensor level CSD to reconstruct the source level CSD of all combination of pairwise voxels. Hence, DICS directly estimates the interaction between sources at respective frequencies. The weight function can be written as

(7) $$W_{S}(f)=\frac{L^{T}C^{-1}(f)}{L^{T}C^{-1}(f)L}.$$

#### eLORETA

eLORETA (Pascual-Marqui, 2007) combines the lead-field normalization with the 3D Laplacian operator under the constraint of smoothly distributed sources. Compared to DICS and LCMV, where the constraint equation $W_{S}\dot{}L^{T}=1$ is used, eLORETA seeks to minimize the product $H=W_{S}\dot{}L^{T}$.

(8) $$W_{S}={\left[L^{T}{\left(LC^{-1}L^{T}+aH\right)}^{†}L\right]}^{1/2},$$

where *a* is regularization parameter, and † is the Moore–Penrose pseudo-inverse, which is equal to the common inverse if the matrix is non-singular. **H** is also called the centering matrix or the surface Laplacian. Low-resolution imaging results in weak performance for recovering of multiple sources when the point-spread functions of sources overlap. Other methods have also tried to combine surface Laplacian with LCMV (Murzin et al., 2013), to estimate source-level connectivity.

#### MNE

MNE has been a popular choice to localize evoked activity and tracking the distribution of the activations over a period of time. MNE is a distributed inverse solution that discretizes the source space into locations on the cortical surface or in the brain volume using a large number of equivalent current dipoles. It estimates the amplitude of all modeled source locations simultaneously and recovers a source distribution with minimum overall energy that produces observed sensor data consistent with the measurement (Hämäläinen and Ilmoniemi, 1994; Ou et al., 2009). The current density *q* can be calculated as

(9) $$q=[CL^{T}{\left(LCC^{T}+\lambda C\right)}^{-}1)].$$

### Measurements used for face validity of inverse algorithms

Using simulated data to test a method provides mechanism for ground truth validation. The exact location of a putative dipolar source is elusive in nature for real data, however, one can certainly set-up simulations when performance of a particular method needs evaluation. We employed three complementary measures to provide face-validity of the eLORETA in comparison with LCMV and DICS.

#### Localization error estimation

Inverse methods estimate a cluster of point dipolar sources. To measure how much error is involved in source localization, we first computed the z-scores for all voxels. Further, we thresholded the z-scores at 99.99th percentile and identified the cluster closest to the dipole location. Then we measured the Euclidean distance between the voxel with the maximum z-score of the nearest cluster source points and the actual source/dipole coordinates, to give us the localization error. This was done for each hemisphere, separately. Consequently, the net localization errors are computed by summing up across two hemispheres and compared for eLORETA, MNE, LCMV, and DICS.

#### Degree of focal localization

The size of a cluster in terms of sum of distances of all points from the voxel with the maximum z-score, gives a measure of focal localization of sources. After finding the z-scores of all voxels, we thresholded the scores at 99.99th percentile and identified the cluster of source points closest to the dipolar location. Further, we computed the total sum of distances of each voxel in the nearest cluster, from the voxel in the cluster with the maximum z-score, as a measure of the spatial distribution of the estimated source. This gave us a quantitative approach to evaluate the degree of focal source localization. For practical reasons, cluster width computation was done for each hemisphere separately (Murzin et al., 2013).

#### Performance evaluation at various depths

The localization of deep sources has been the main factor limiting detection of true sources from MEG/EEG data. This is important, since deep cortical areas constitute 30% of the cortical sources (Hillebrand and Barnes, 2002). We studied the effects of depth by positioning the dipoles at different distances from the auditory cortical locations mentioned earlier. The depth was varied along the *x*-axis, from 0 to 20 mm in steps of 1 mm, towards the center of the brain, in both hemispheres. Further, we computed the localization error and the focal width of the significant voxels obtained by localizing the dipoles placed at each depth. This was executed using the distributed dipolar method only. All simulated signals were added to the empirical baseline to retain the physiological SNR for the Gaussian pulses and the physiological power spectrum for the sinusoidal. The signals simulated were phase-locked (sinusoidal) and no time lags were added (Gaussian)

#### Performance evaluation with various correlation

Correlation in the data covariance is an important variable which can influence localizing capabilities of beamformers (Belardinelli et al., 2012). Therefore, we simulated multiple signals using the distributed dipolar model, consequently adding with the empirical baseline, such that there are various phase lags between the signal simulated in the left hemisphere and the right hemisphere. Four phase lags chosen for frequency domain analysis were 0, *π*/2, *π*, 3*π*/2. The power ratios were matched as per the acquired empirical power ratios. Four time lags chosen for time domain analysis were: 0, 15, 30, 45 ms.

#### FP percentage

We compared the “sensitivity” and “specificity” of eLORETA, LCMV, and DICS, using ROC analysis (Metz, 1978). Here, we calculated the probability of incorrectly detecting an activation, also called FP. Ideal detection should suppress FP. After thresholding the z-scores, we identified the number of significant clusters in each hemisphere, visually. Further we ran k-means clustering over significant voxel locations in each hemisphere and identified the nearest cluster to the true dipole location. Defining the nearest significant cluster/s from the dipolar location/s as the true positive/s, we further defined the FP percentage by computing the ratio of number of significant voxels not present in the true positive (or nearest cluster) and the total number of significant voxels. We also compare the performance of all methods under parametric variation of SNR at the source level and different kinds of source configurations, e.g., single-point dipole, two-point dipoles, and distributed dipoles.

#### Hit rate

To evaluate the accuracy of localizing algorithms, we computed the hit rate or true positives for each method. After thresholding the z-scores and obtaining the significant clusters (using k-means), we identified the number of significant source points in the nearest cluster within a distance of 15 mm from the simulated dipole location. These source points were defined as hits. The hit rate corresponds to the ratio of number of hits and total number of significant source points in the nearest cluster. Hit rates were calculated across all SNRs and power ratios, including lags and different phase differences, respectively, for single-point, two-point, and distributed dipoles.

### Code accessibility

All codes used for simulation of data and source localization algorithms is available at the following GitHub repository https://github.com/arpan-toolboxes/QuantitativeSourceImaging. The reader is encouraged to contact the authors in case of implementation issues.

### Empirical EEG recordings

#### Participants

Ten healthy volunteers (eight males, two females) aged between 22 and 39 years (mean 28 years old) participated in the study after giving informed consent, following the guidelines approved by Institutional Human Ethics Board at National Brain Research Center. All participants were self-declared normal individuals with no history of hearing impairments and had either correct or corrected-to-normal vision and no history of neurologic disorders.

#### Stimuli

Volunteers had to remain stationary in a seated position within a sound-proof room and hear auditory stimuli through 10-Ω insert earphones with disposable foam ear-tips, binaurally for 200 s while fixating at a visual cross. Additionally, they had a baseline block where they fixated at the visual cross for 200 s without any sounds being played. Sounds were pure tones of 1000-Hz frequency and 25-ms time duration, with 5% rise and fall times and were repeated with a frequency of 40 Hz during an ON block of 1-s duration interspersed between two OFF blocks where no auditory stimuli were presented. Stimuli were made using in STIM2 stimulus presentation system with audio box P/N 1105 at 85 dB.

#### Data collection and pre-processing

EEG data were acquired in an acoustically shielded room with 64 channels NeuroScan (SynAmps2) system with 1-kHz sampling rate. Brain Products abrasive electrolyte gel (EASYCAP) was used to make contact with scalp surface and the impedance was maintained at values less than 5 kΩ for all volunteers. Baseline EEG data were recorded for 200 s with eyes open, no tone, and a fixation cross on a monitor in front of the participants. Baseline and binaural stimuli were presented while participants were asked to maintain fixation on the cross all along to reduce eye movements.

Recorded raw data were re-referenced with average reference and were detrended to remove linear trends from the signal. Epochs of 5-s duration were constructed by concatenating ON blocks of 1 s each after removal of an initial 50 s of the 200-s-long session. This was done to capture ASSRs. Data were band pass filtered with cutoff frequencies 5–48 Hz, to concentrate on sources underlying ASSR.

For an evoked wave form analysis, after average re-referencing, epochs of 1-s duration of ON blocks were extracted from the raw data during stimulus condition, then filtered with cutoff frequencies 0.5–48 Hz, detrended, and averaged across trials to generate the evoked potential. Thresholds of –100 and 100 μV were used to reject blink-corrupted trials, meaning if at any point within the epoch the voltage exceeded the threshold values, the entire trial was deleted from the subsequent analysis.

Sensor locations were taken from the template given in the fieldtrip toolbox. Colin 27 structural T1 was used for co-registration with the sensor locations for accurate source localization. A forward model was computed using boundary element method (BEM) from the respective T1 image. For localization using the algorithms, we considered 0% regularization for all methods. The ratio of source power between stimulus and baseline condition was calculated in each voxel, using (*Power*(*Stimulus*)–*Power*(*Baseline*))./*Power*(*Baseline*) for the current density measures and (*Power*(*Stimulus*)–*Power*(*Baseline*)) for the beamformers. After computing the source intensities in each volunteer, the individual grids were interpolated to the T1 image. The averaged voxel intensities across all participants were evaluated using non-parametric statistics, and z-scores were computed for each hemisphere. The top 0.05% voxels were identified as sources.

## Results

### Simulated EEG data

Simulated EEG data were computed by placing electric dipolar sources at auditory cortical locations, according to single dipole, two-point dipoles and distributed dipole configurations, using Equation 1 and projecting the source activity at realistic sensor locations of a Neuroscan (Compumedics Inc) EEG cap using a realistic head model (Equation 2; Baillet et al., 2001). We considered two types of temporal profiles for source activity, a sinusoidal signal mimicking the band-specific frequency response observed in typical EEG signal such as ASSR and a mixture of Gaussian pulses representing the time-locked ERPs. Baseline data were acquired empirically on which both the aforementioned signals were added. Two prototype examples of simulated scalp activity during task and baseline are illustrated in Figure 1. To observe the effects of correlation in the CSD matrix on source localization, phase lags were introduced to the simulated sinusoidal signals generated from each hemisphere (two-point and distributed dipoles), as well as, time delay was added to the Gaussian pulses to the signal from the right hemisphere in two-point and distributed models.

**Figure 2.**
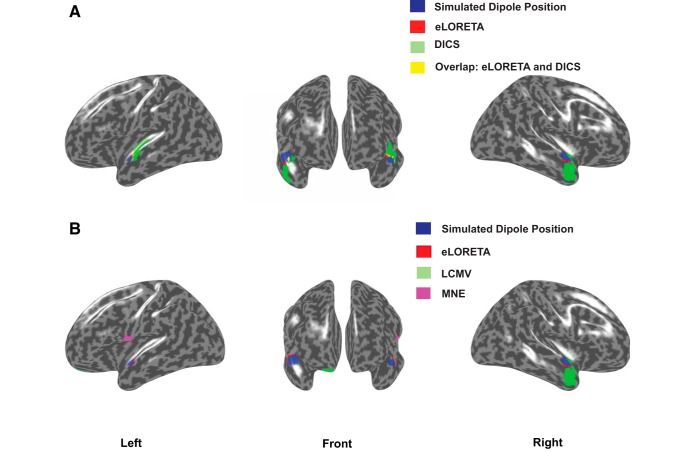
***A***, eLORETA (red) versus DICS source localization (green) using frequency-lock analyses on distributed dipolar source generated signals. Overlapping regions from both analyses are depicted in yellow and locations used for simulated dipole placements are depicted in blue. eLORETA was applied to the simulated sinusoidal signal embedded in resting state EEG, where the simulated signals generated from each hemisphere had 0 phase lag. For DICS, the simulated signal from the right hemisphere had a phase lag of *π* with respect to the signal from the left hemisphere. The ratio of power spectrum at 40 Hz between the sinusoidal embedded in EEG and resting state EEG was chosen similar to the ratio found in our empirical results. ***B***, eLORETA (red), LCMV (green), and MNE (purple) generated sources using time-lock analysis. No overlapping regions were found. The results indicate 0 time-lag scenario between the Gaussian pulses from each hemisphere. Physiologically realistic SNR 25 dB for simulated dipolar sources was chosen for this illustration.

We applied eLORETA and DICS to perform frequency-locked source analysis on the sinusoidal data. Keeping the empirical baseline intact, we scaled the sinusoidal signal such that, we obtained different ratios of power of the sinusoidal at 40 Hz, comparable to the values we found in our empirical data. Figure 2*A*, illustrates combined results from eLORETA and DICS algorithms on a brain surface rendered by the MNI brain, at power ratio of 1 (realistic SNR), for the distributed dipole model.

The algorithms were employed for localizing the sources of the peak negative response in mixture of Gaussians signal, by selecting a time segment constituting of points ±25 ms around the peak (Fig. 1*B*). For plotting activations, the source locations in the 3D voxel space was projected to a surface plot using customized MATLAB codes.

The localization error was computed by first defining each voxel as point in a cluster and thereby determining the distance of the voxel with the maximum z-score from the true dipole source in each hemisphere. For distributed dipolar sources, the center of the spherically distributed source was considered as the true dipole location. The average of sum of distances from all such points to the voxel with the maximum z-score, normalized by the total number of points was used to quantify the focal localization of sources, in each hemisphere. All voxels were then transformed to their nearest projections on the cortical surface and identified as possible source locations in Figure 2. The quantitative evaluation of the performances of the inverse methods are addressed as follows.

#### Accuracy

**Figure 3.**
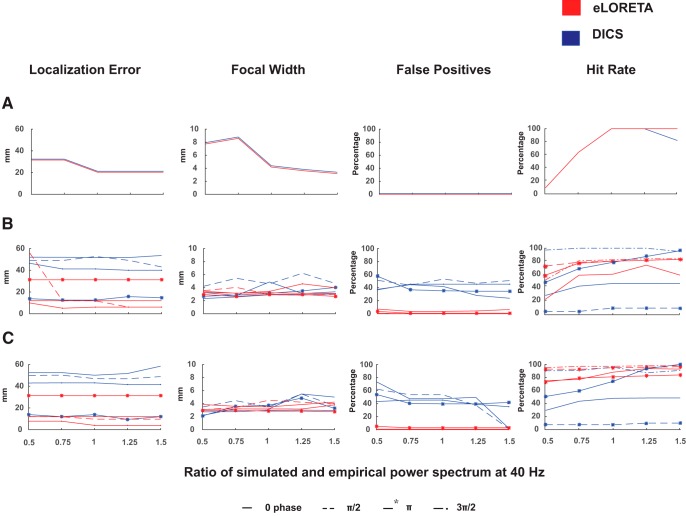
Localization error, focal width, FP, and hit rate percentage for (***A***) single dipole, (***B***) two-point dipole, and (***C***) distributed dipole conditions measured for eLORETA (red) and DICS (blue). Source localization was done for all power ratios (*x*-axis) across different simulated phase lags of 0, *π*/2, *π*, and 3*π*/2.

Frequency analyses using eLORETA and DICS yielded similar localization errors with respect to different power ratios, in one dipole condition giving 0 FPs (Fig. 3). However, eLORETA provided much lower localization error than DICS for two-point and distributed dipole conditions. This was observed at lags: 0, *π*/2 , and 3*π*/2, where DICS performed comparatively poorly. Interestingly, DICS performed better than eLORETA at phase lag of *π* in terms of accuracy, at which eLORETA’s accuracy deteriorated. Overall, the most significant observation was linked to the consistent performance of the algorithms regardless of the power spectra at 40 Hz.

**Figure 4.**
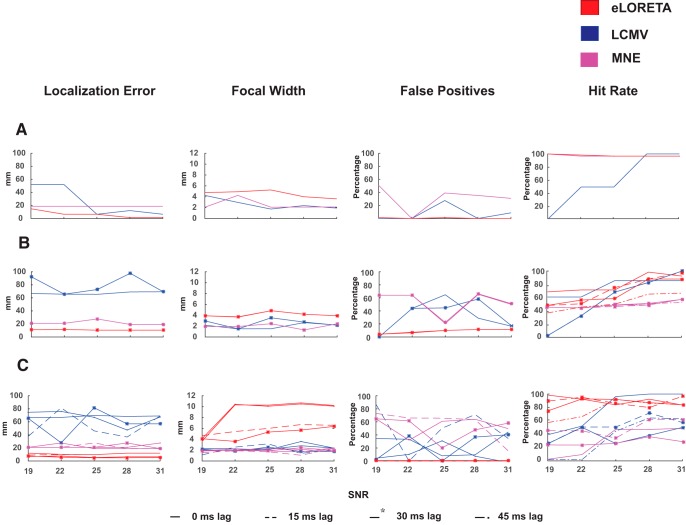
Localization error, focal width, FP, and hit rate percentage for (***A***) single dipole, (***B***) two-point dipole, and (***C***) distributed dipole condition measured for eLORETA (red), LCMV (blue) and MNE (pink). Source localization was done for all SNRs (*x*-axis) across different simulated time lags where 25 dB refers to the realistic scenario.

In time domain analysis, eLORETA performed better in all dipole configurations in contrast to MNE and LCMV (Fig. 4). eLORETA consistently provided localization error of around 1 cm or less for all time lags. LCMV’s accuracy with respect to SNR’s and time lags was not consistent as no trend could be observed. In contrast, MNE was observed to be better than LCMV at almost all scenarios with consistent errors across all SNRs. Therefore, it can be pointed that SNR and time lags affect the performance of the beamformers and minimal effects can be found in the current density measures, in terms of accuracy.

#### Localization spread

eLORETA performance is observed to be comparable (similar focal localization) to DICS across all dipole conditions as well as power ratios (Fig. 3). Apart from slightly higher values of focal localization for DICS at *π*/2 lag, both algorithms are efficiently focal.

The focal localization of eLORETA on time-locked signal was observed to reduce for single- and two-dipole condition, across different SNRs, however, not varying across time lags (Fig. 4). MNE and LCMV performance was comparable and also did not vary across time lags. However, the focal width of eLORETA deteriorated in the distributed dipole condition and varied heavily across time lags, in contrast to unperturbed performance of LCMV and MNE. Additionally, the focal width of eLORETA increased with increasing SNR. However, it was noted that there was minimal effect of SNR on the focal width of the other algorithms.

#### Depth

**Figure 5.**
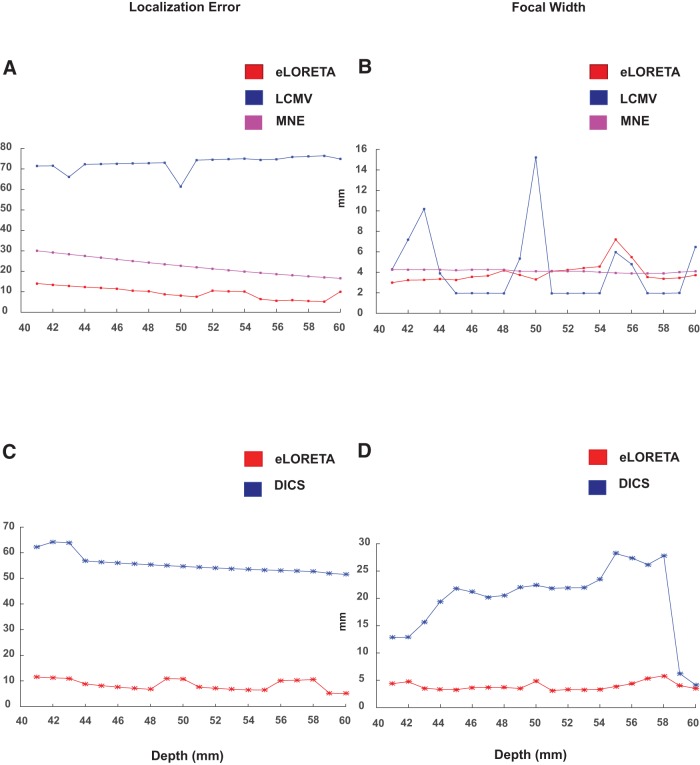
Localization errors and focal widths of all significant voxels computed for simulated distributed dipoles with the center of the dipole cluster located at different depths from the auditory cortical locations. The depth of the dipoles decreases along the *x*-axis; the distance from the auditory cortex. ***A***, ***B***, Localization error and the focal width of the sources generated by the mixture of Gaussian signals, respectively. ***C***, ***D***, exhibit the localization error and focal width for frequency domain analysis.

To quantitatively measure the localizing capabilities of eLORETA, DICS, MNE, and LCMV, source localization was executed for distributed dipoles at different depths. The localization error and the focal width were computed for the significant voxels, illustrated in Figure 5. The depth of dipolar locations was varied along the *x*-axis according to the MNI template, 1 mm apart for each localization iteration. The deepest positions were selected as [–40, –28, 6] in the left hemisphere and [40, –24, 6] in the right hemisphere. The locations closest to the surface were chosen as [–60, –28, 6] in the left hemisphere and [60, 28, 8] in the right hemisphere.

The localization error for DICS declined and performed favorably as the depth of the dipoles decreases. A difference of 10 mm was observed between the deepest and the most superficial source for DICS. However, eLORETA gave consistent low localization errors across all depths. It is observed to be more focal than DICS across all depths, except for comparable focal width for superficial sources.

For the time-locked condition, the current density measures performed better than the beamformer, for localizing the Gaussian signal at all depths (performance of eLORETA>MNE>LCMV). Although LCMV was found to be more focal at certain depths, eLORETA and MNE were found to be focal across varying depths. eLORETA proved to be slightly more focal than MNE at deeper depths, in contrast to MNE being more focal the eLORETA at smaller depths. Therefore, eLORETA can be credited to have a higher degree of focal localization in comparison to MNE, LCMV, and DICS, for localizing sinusoidal, as well as Gaussian, pulse, especially at deeper depths.

#### FP percentage

To evaluate specificity, we computed the FPs after applying DICS, LCMV, and eLORETA in different SNR scenarios, for all phase/time lags across different dipole conditions. All source points except the cluster of points nearest to the dipole location were considered as FPs (for details, see Materials and Methods, Measurements used for face validity of inverse algorithms).

With 0 FPs for one-dipole condition, the FPs of DICS increased with increasing number of dipoles (Fig. 3). With the percentage ranging from 30–70%, eLORETA provided a very high rate of true positives across all SNRs and phase lags. Unsurprisingly, eLORETA gave a very low FP fraction on localizing the Gaussian, similar to localizing the sinusoidal (Fig. 4). MNE and LCMV gave high and comparable but consistent FPs fraction across all time lags in single- and two-dipole condition. However, in the distributed dipole case, the FPs of MNE and LCMV increased with varying time lags.

Table 1 classifies the performance of all the methods based on accuracy, localization, and sensitivity across all dipole conditions for localizing 40-Hz sinusoidal signal (frequency analysis) and Gaussian pulse response (time lock), respectively.

**Table 1.** Outcome of source localization performance based on different metrics for frequency analyses (eLORETA, DICS) and time domain analyses (eLORETA, LCMV, MNE)

Frequency analysis			
**Dipole condition**	**Localization error**	**Focal width**	**False positives (FPs)**
Single dipole	eLORETA/DICS	eLORETA/DICS	eLORETA/DICS
Two-point dipoles	eLORETA(0, *π*/2, 3*π*/2)/ DICS(*π*)	eLORETA	eLORETA
Distributed dipoles	eLORETA(0, *π*/2, 3*π*/2)/ DICS(*π*)	eLORETA	eLORETA
Time-lock analysis			
Single dipole	eLORETA	MNE/LCMV	eLORETA
Two-point dipoles	eLORETA	MNE/LCMV	eLORETA
Distributed dipoles	eLORETA	MNE/LCMV	eLORETA

If similar performance was achieved, both methods are mentioned with “/.”

#### Hit rate

eLORETA and DICS proved to be reliable in localizing single dipole, by yielding 100% hit rates at SNRs of biological level and higher. However, DICS indicated varied results with respect to the different power ratios at 40 Hz between the simulated and empirical data. In both, distributed and two-point dipolar model as the hit rates plummeted at phase difference of *π*. In contrast, eLORETA had high hit rates irrespective of phase differences and further increased with higher number of dipoles. For algorithms localizing the temporal features of the signal, the beamformer LCMV’s performance was influenced by SNR, i.e., hit rates increased with increasing SNR’s across all models. However, this was not true for the current density measures, since they were minimally influenced by SNR. Despite almost perfect localization of single dipole by current density measures, eLORETA yielded higher hit rates compared to MNE in two point and distributed dipole models. Similar to DICS, the hit rates corresponding to LCMV varied with respect to different temporal lags. To summarize, eLORETA’s hit rates were superior among beamformers and current density measures.

### Empirical EEG data

#### Source localization underlying 40-Hz EEG activity

**Figure 6.**
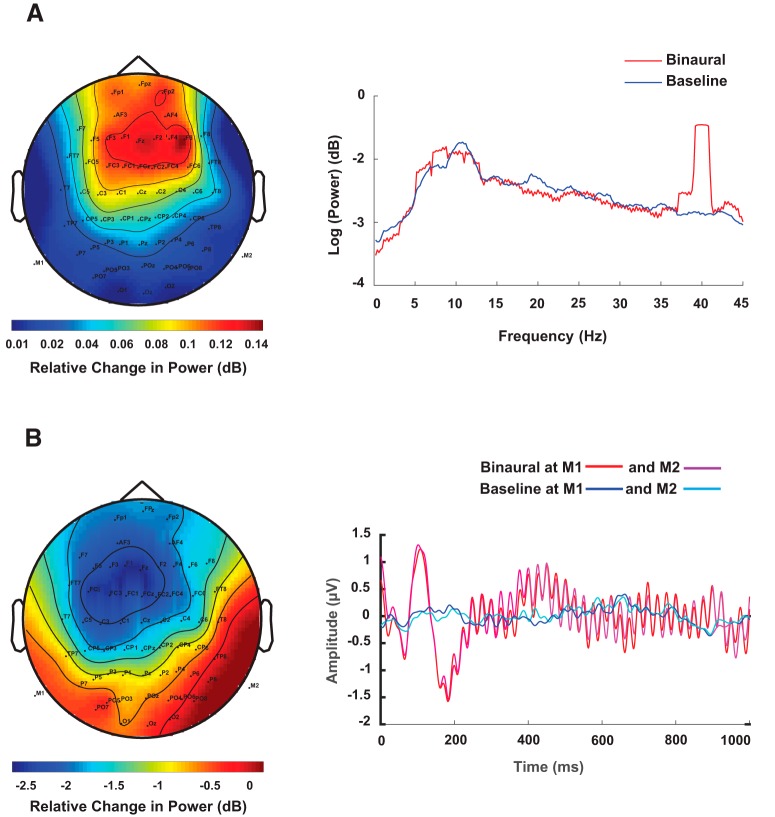
***A***, Topoplot of spectral power difference at 40 Hz and grand average of spectral power across all sensors, trials, and participants in binaural and the baseline conditions. Power spectral density was calculated for 5-s windows after rejecting an initial 50 s out of total duration of 200 s for which the rhythmic tones were played. ***B***, ERP responses of channels M1 and M2 across trials and participants for binaural and silent baseline conditions and the topoplot for the difference signal at the peak of N100 response (at 110 ms).

**Figure 7.**
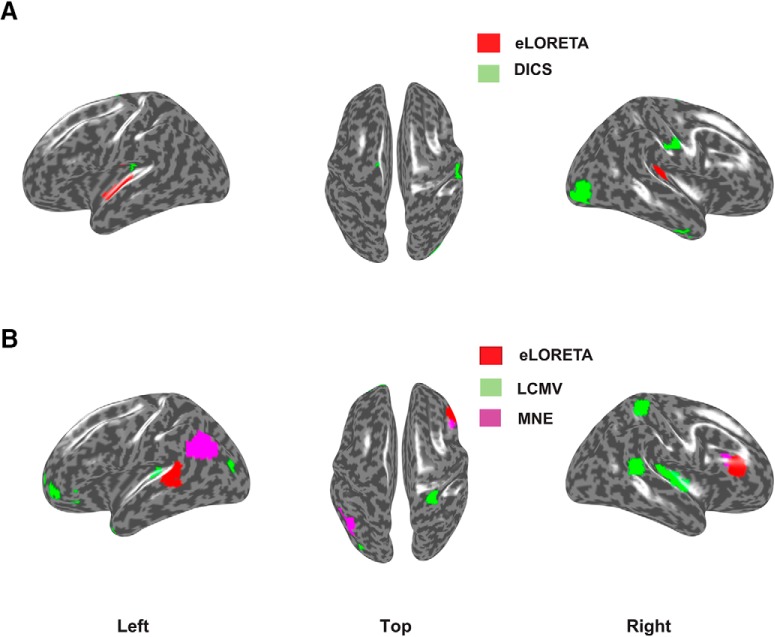
Left, Top and right view of significantly active cortical sources underlying for (***A***) 40-Hz ASSR and (***B***) N100 component of the ERP. The red colored regions show estimated sources from eLORETA while green regions show estimated sources from (***A***) DICS and (***B***) LCMV analysis. The pink regions in ***B*** show MNE source localization results.

The Fourier spectrum of each EEG channel time series was computed by multi-taper method with number of tapers = 2, using Fieldtrip function ft_freqanalysis.m. Power spectral density of empirical EEG data and the ERP time locked to the onset of a single tone stimulus are shown in Figure 6. In Figure 6*A*, the topoplot of the difference in power between binaural and baseline conditions at 40 Hz is shown along with the log of power across all trials. There are peaks at alpha band 8–12 Hz in both binaural and baseline conditions the difference between which was not significant (two-sample *t* = –0.018, *p* = 0.95), whereas the binaural condition had a sharp rise in power at 40 Hz, which was significant (two-sample *t* = 0.18, *p* < 0.0001). The *t* tests were performed on logarithm of power spectrums from two conditions within a frequency bands, 8–12 Hz for alpha and 39.8–40.2 Hz for evoked 40 Hz. Localization results are illustrated in Figure 7*A*. The left, top, and right views of activation are shown where red regions represent sources from eLORETA analysis while green regions represent activation plots generated by DICS.

Acknowledging the absence of ground truth concerning true sources that exist in empirical data, we evaluated the focal width of all the significant clusters after thresholding the z-scores. One such cluster was found in each hemisphere around the auditory cortex for eLORETA, providing a mean focal width of 3.958 mm. In contrast, DICS yielded one cluster at the auditory cortex in the left hemisphere and seven distributed clusters in the right. The mean focal width across DICS clusters was 2.8324 mm, lesser than that of eLORETA. Understanding that the thresholding can vary focal width and number of clusters, we maintained the same threshold for both the algorithms for a fair comparison. It is to be noted that the mean focal width of two clusters of eLORETA can be decreased with higher thresholding.

#### Source localization of N100 response

In Figure 6*B*, we show the ERP responses to the binaural tone and the ERPs in baseline condition, averaged across all trials and participants (grand average). A negative peak around 100 ms after onset of tone stimulus (N100) was observed in the binaural condition with a latency of around 110 ms. The topoplot represents the spatial map of the difference in relative changes of amplitude between ERPs from the binaural and baseline conditions across all channels and trials.

Next, we computed the underlying source activation during the N100 response using LCMV and eLORETA. In Figure 7*B*, we plot the source activations (top 0.05% voxels similar to 40-Hz case) in epochs of duration 50 ms, within which the 25th millisecond corresponds to the peak of N100. The beamformer localized bilateral auditory cortices, along with other distributed significant clusters. It can be noted that LCMV may localize the underlying activity, however, has higher probability of yielding FPs, due to its distributed activations. In contrast, the current density measures localized the left auditory cortex (eLORETA) and left posterior superior temporal sulcus (MNE). Computing only one cluster for each hemisphere, eLORETA and MNE yielded a significant cluster in the right frontal regions. The focal width of LCMV (2.1706 mm) was lesser than current density measures (3.1–3.3 mm), similar to the case in the frequency domain analysis.

## Discussion

Identifying the sources underlying key events of information processing such as ERP peaks or oscillatory brain activity such as spontaneous gamma oscillations are the objectives of many research studies. However, different inverse methods provide different solutions leading to no agreement of which algorithm is the “best method,” as we illustrated in this manuscript with simulated and empirical EEG data. Although the selection of the best method can be guided by the nature of the hypothesis in a putative experimental design, a systematic comparative account of the efficacies of few prominent methods is currently missing in the literature. To address this issue we compared methods, eLORETA (Pascual-Marqui, 2007), LCMV (Van Veen et al., 1997), MNE (Hämäläinen and Ilmoniemi, 1994), and DICS (Gross et al., 2001) using the metrics that evaluates accuracy, sensitivity, and specificity across three dipolar models (single, two-point, and distributed) for the simulated sinusoidal signal (mimicking the steady state 40 Hz) and a mixture of Gaussian pulses (representing the time locked ERP). Furthermore, we chose an empirically observed baseline which is a key ingredient for every inverse method. The models were simplistic, however, since we knew the exact location of dipole/s, ground truth validation was possible. All methods are able to retrieve the location of the true dipolar sources for a physiologically relevant SNR and frequency power ratio (Fig. 2, blue areas). Nonetheless, the study was conducted across different SNRs and frequency power spectra to test each method’s sensitivity and specificity to noise. Furthermore, performance of all methods was evaluated with respect to dipolar depth, phase lags among sources, accuracy and localization spread over space. Also, we conducted source localization by collecting empirical EEG data exhibiting 40-Hz ASSR (Fig. 7). DICS and eLORETA were used to compute the sources underlying the 40-Hz activity, and LCMV and eLORETA were used to compute the sources underlying the N100 response (Fig. 7). Thus, we could outlay the hallmarks of each method in an organized framework. The key finding of our study is that although high hit rates and focal localization are achieved with both current density and beamformer approaches, the FPs and focal width of sources needs to be carefully considered while choosing a specific method, and this is where eLORETA scores above most of the other methods.

A general consensus emerges from comparing the algorithms that there is no clear winner (Table 1) if accuracy as well as sensitivity and specificity are all taken together as guiding parameters. DICS gives better accuracy than eLORETA in single- and two-point dipole conditions even at low SNRs; however, the focal width of eLORETA generated sources is always slightly better most likely due to the minimization of the surface Laplacian component while estimation of the spatial filter. For distributed dipole scenario, focal width of eLORETA results were very similar to DICS, in fact getting better with higher SNR. Interestingly, eLORETA shows significant control on the FP ratio in the distributed dipole condition, proving to be the method of choice for estimating sources underlying frequency response in a more exploratory setting. This is indeed a very important point to note for increasing number of studies studying resting state functional connectivity (Canuet et al., 2011; Custo et al., 2017). Once a hypothesis is put in place with some prior knowledge about the involvement of prospective brain networks, one can go for DICS that can produce more accurate results (Tan et al., 2016). Interestingly, DICS results in lowest localization error with maximum phase lag of *π*, whereas the effect was reverse for eLORETA.

For source localization of ERP peaks, eLORETA majorly yielded better accuracy and specificity (in terms of favorable FPs) than LCMV and MNE, although the focal width of eLORETA sources were considerably larger than LCMV and MNE. The pitfalls and advantages of each method are summarized in Table 1. As in the case of frequency domain analysis, we would recommend eLORETA for an exploratory level analysis, whereas LCMV or MNE for more hypothesis-driven identification of sources.

An alternative solution to increase the probability of isolating an active source is to combine two or more methods and take the overlap of sources detected from them as the plausible source configuration. We provide a blueprint of these using our empirical and simulated data. Both spectral domain eLORETA and DICS were able to pinpoint the left auditory cortex, the location of one true source (Fig. 2). The number of FPs obtained with two methods combined is drastically low. This gives us the confidence that ASSR involves strong activity in primary sensory regions of auditory processing, e.g., bilateral middle temporal gyrus as reported by earlier studies (McFadden et al., 2014; Tan et al., 2016) and as we observe in Figure 7. However, for current empirical data, we did not find overlapping regions from different techniques, validation from two or more methods will give a strong confidence in the result of source localization. Proof-of-concept illustration is available for time-lock analysis as well (Fig. 7), where we present combined eLORETA, LCMV, and MNE to identify the sources underlying N100 peak.

In future, we think an overlap-approach might result in focal localization with minimum number of FPs. This will particularly benefit the identification of sources whose activity may be relevant for a particular context. For example cross-frequency coupling (CFC) between alpha and gamma rhythms are being postulated to be important for gating of attention (Klimesch, 2012). How to identify a cortical subnetwork whose nodes show CFC out of the whole alpha and gamma networks is an important methodological challenge. We believe a conjunction of methods strategy to identify the potential sources will be crucial from the perspective of reliability as well as accuracy. In summary, our study provides a blue print for employing source-localization techniques to isolate more subtle features of signal processing.

## Synthesis

Reviewing Editor: Gustavo Deco, Universitat Pompeu Fabra Decisions are customarily a result of the Reviewing Editor and the peer reviewers coming together and discussing their recommendations until a consensus is reached. When revisions are invited, a fact-based synthesis statement explaining their decision and outlining what is needed to prepare a revision will be listed below. The following reviewer(s) agreed to reveal their identity: Theodore Zanto.

Reviewer 1:

This manuscript addresses a topic that is important for EEG research. It compares various existing EEG inverse solutions methods for the accuracy of localization of sources (e.g. with location error, focal width, depth and false positives, etc). The conclusions seem to suggest that one should use some combination of multiple source-localization methods for reliability and accuracy.

Although the article is well written and the analysis has been carried out systematically, they do not arrive at a clear conclusion about the usage. The conclusion also seem to be at odds with with the results about eLORETA method. eLORETA is known for zero localization error: http://www.uzh.ch/keyinst/eLORETA/.

Reviewer2:

The manuscript "Quantitative evaluation of reliability in estimating sources underlying brain oscillations using current source density methods and minimum norm estimates" assesses multiple source localization methods of frequency and time-based EEG signals - using both simulated and recorded data. Results highlight strengths and weaknesses of each approach and the authors provide recommendations for the use of each approach. The article is very straightforward and provides practical knowledge for the MEG/EEG community. I only have a few minor concerns and comments detailed below.

I have two comments about the title. 1) Because the term "reliability" usually refers to the ability to get the same results across subjects or across days (within a subject), it would make sense to remove "of reliability" from the title. 2) In the text, you note that you're evaluating "the performance of the key current density techniques: eLORETA, MNE and beamformer approaches: DICS and LCMV". As such, shouldn't the title replace "minimum norm estimates" for "beamformer approaches"? Together, I would suggest the title, "Quantitative evaluation in estimating sources underlying brain oscillations using current source density methods and beamformer approaches."

While the Localization Error, Focal Width and False Positives are great measures of performance, it would be useful to understand the Hit Rate as well. In other words, how many of the dipolar locations were detected by each algorithm (as a percentage)? This way, a reader may look at the hit rate and false positive rate and make a more informed decision about the costs and benefits of each type of method.

The colors in Figure 2 are not fully described. What does blue and yellow represent? I assume blue is where the 2 methods overlap? Though the discussion section makes me think blue is where the source is actually located. Please clarify - and indicate where the source was simulated, as well as where both methods overlap and where they overlap with the source. You can use a different color for each scenario. For example, in Figure 2a, red could be eLORETA only (i.e., not overlapping with the source or DICS), green could be DICS only (not overlapping with the source or eLORETA), purple could be where both eLORETA and DICS overlap, but does not overlap with the source, blue could represent where both methods overlap in the source region, and yellow could be the source only (no method localized to that region). A similar coloring scheme could be extended to Figure 2b where MNE is also included.

Similarly, Figure 7 could benefit from colors indicating overlap between methods.

Various grammatical errors, for example:

Line 152, "-scores" should read "z-scores"

Line 156, "hemsiphere" should read "hemisphere"

Line 177, you appear to question whether your statement is correct, "...which can influence localizing capabilities of beamformers (?)."

## Author Response

We are grateful to the anonymous reviewers for positive consideration and suggestions to improve the manuscript. In the revised manuscript we have addressed the concerns as follows.

Reviewer 1:

This manuscript addresses a topic that is important for EEG research. It compares various existing EEG inverse solutions methods for the accuracy of localization of sources (e.g. with location error, focal width, depth and false positives, etc). The conclusions seem to suggest that one should use some combination of multiple source-localization methods for reliability and accuracy.

Although the article is well written and the analysis has been carried out systematically, they do not arrive at a clear conclusion about the usage. The conclusion also seem to be at odds with with the results about eLORETA method. eLORETA is known for zero localization error: http://www.uzh.ch/keyinst/eLORETA/.

Thank you for the comments and suggestions.

We agree with the observation that our main message is that there exist no clear winner in terms of source localization technique. All have their merits and pitfalls. An effort has been made to suggest the method of choice for appropriate context in Table1. We highlight am earlier statement about the need for this paper in blue in Discussion.

“However, different inverse methods provide different solutions leading to no agreement of which algorithm is the `best method', as we illustrated in this manuscript with simulated and empirical EEG data. Even though the selection of the best method can be guided by the nature of the hypothesis in a putative experimental design, a systematic comparative account of the efficacies of few prominent methods is currently missing in the literature. To address this issue we compared methods, eLORETA (Pascual-Marqui, 2007), LCMV (Van Veen et al., 1997), MNE (Hamalainen and Ilmoniemi, 1994) and DICS (Gross et al., 2001) using the metrics that evaluates accuracy, sensitivity and specificity across three dipolar models (single, two-point and distributed) for the simulated sinusoidal signal (mimicking the steady state 40 Hz) and a mixture of Gaussian pulses (representing the time locked ERP). Furthermore, we chose an empirically observed baseline which is a key ingredient for every inverse method.”

The eLORETA claim about exact localization relates to recovery of all dipoles, but a detailed discussion of false positives is missing in the literature. In response to the aforementioned comment we added the following sentences in abstract :

“All methods were successful in recovery of point sources in favorable signal to noise scenarios and could achieve high hit rates if false positives are ignored. Interestingly, focal activation map is generated by LCMV and DICS when compared to eLORETA while control of false positives and hit rates is much superior in eLORETA. Subsequently drawbacks and strengths of each method are highlighted with a detailed discussion on how to choose a technique based on empirical requirements.”

And in Discussion section

“The key finding of our study is that even though high hit rates and focal loclization are achieved with both current density and beamformer approaches, the false positives and focal width of sources needs to be carefully considered while choosing a specific method and this is where eLORETA scores above most of the other methods.”

______________________________________________________________________

Reviewer2:

The manuscript “Quantitative evaluation of reliability in estimating sources underlying brain oscillations using current source density methods and minimum norm estimates” assesses multiple source localization methods of frequency and time-based EEG signals - using both simulated and recorded data. Results highlight strengths and weaknesses of each approach and the authors provide recommendations for the use of each approach. The article is very straightforward and provides practical knowledge for the MEG/EEG community. I only have a few minor concerns and comments detailed below.

Thank you for the positive comments

I have two comments about the title. 1) Because the term “reliability” usually refers to the ability to get the same results across subjects or across days (within a subject), it would make sense to remove “of reliability” from the title. 2) In the text, you note that you're evaluating “the performance of the key current density techniques: eLORETA, MNE and beamformer approaches: DICS and LCMV”. As such, shouldn't the title replace “minimum norm estimates” for “beamformer approaches”? Together, I would suggest the title, “Quantitative evaluation in estimating sources underlying brain oscillations using current source density methods and beamformer approaches.”

Agreed and title changed.

While the Localization Error, Focal Width and False Positives are great measures of performance, it would be useful to understand the Hit Rate as well. In other words, how many of the dipolar locations were detected by each algorithm (as a percentage)? This way, a reader may look at the hit rate and false positive rate and make a more informed decision about the costs and benefits of each type of method.

We have included hit rate as another metric, to evaluate the accuracy of the algorithms. We have made edits in the method section, describing how we measured the hit rate, as well as, in the results and discussion. Overall, we have observed higher hit rates with current density measures compared to beamformers.

The colors in Figure 2 are not fully described. What does blue and yellow represent? I assume blue is where the 2 methods overlap? Though the discussion section makes me think blue is where the source is actually located. Please clarify - and indicate where the source was simulated, as well as where both methods overlap and where they overlap with the source. You can use a different color for each scenario. For example, in Figure 2a, red could be eLORETA only (i.e., not overlapping with the source or DICS), green could be DICS only (not overlapping with the source or eLORETA), purple could be where both eLORETA and DICS overlap, but does not overlap with the source, blue could represent where both methods overlap in the source region, and yellow could be the source only (no method localized to that region). A similar coloring scheme could be extended to Figure 2b where MNE is also included.

Similarly, Figure 7 could benefit from colors indicating overlap between methods.

We have added to the legend in Fig2, stating that the blue cluster as the location of the simulated dipole. We have now depicted the overlapping regions from DICS and eLORETA, Fig 2 (top row), in yellow. However, for time-locked analysis didn't have any overlapping regions from 3 methods even though from visual inspection the results from eLORETA and LCMV looked close.

No overlapping regions were found in Fig 7. We also have a more nuanced description of the overlap approach in the Discussion section.

“Although, for current empirical data we did not find overlapping regions from different techniques, validation from 2 or more methods will give a strong confidence in the result of source localization. Proof-of-concept illustration is available for time-lock analysis as well (Fig 7), where we present combined eLORETA, LCMV and MNE to identify the sources underlying N100 peak.”

Various grammatical errors, for example:

Line 152, “-scores” should read “z-scores”

Line 156, “hemsiphere” should read “hemisphere”

Line 177, you appear to question whether your statement is correct, “...which can influence localizing capabilities of beamformers (?).”

Thank you for pointing out these mistakes. These along with other such typos corrected. The question mark was a missing citation!
